# Structural insights into the assembly and regulation of distinct viral capsid complexes

**DOI:** 10.1038/ncomms13014

**Published:** 2016-10-04

**Authors:** Subir Sarker, María C. Terrón, Yogesh Khandokar, David Aragão, Joshua M. Hardy, Mazdak Radjainia, Manuel Jiménez-Zaragoza, Pedro J. de Pablo, Fasséli Coulibaly, Daniel Luque, Shane R. Raidal, Jade K. Forwood

**Affiliations:** 1 School of Animal and Veterinary Sciences, Charles Sturt University, Boorooma Street, Wagga Wagga, New South Wales 2678, Australia; 2 Graham Centre for Agricultural Innovation, NSW Department of Primary Industries and Charles Sturt University, Boorooma Street, Wagga Wagga, New South Wales 2678, Australia; 3 Centro Nacional de Microbiología/ISCIII, Majadahonda, Madrid 28220, Spain; 4 School of Biomedical Sciences, Charles Sturt University, Wagga Wagga, New South Wales 2678, Australia; 5 Australian Synchrotron, 800 Blackburn Road, Clayton, Victoria 3168, Australia; 6 Infection and Immunity Program, Monash Biomedicine Discovery Institute and Department of Biochemistry and Molecular Biology, Monash University, Melbourne, Victoria 3800, Australia; 7 Física de la Materia Condensada, Universidad Autónoma de Madrid, 28049 Madrid, Spain; 8 Insituto de Física de la Materia Condensada (IFIMAC), Universidad Autónoma de Madrid, 28049 Madrid, Spain

## Abstract

The assembly and regulation of viral capsid proteins into highly ordered macromolecular complexes is essential for viral replication. Here, we utilize crystal structures of the capsid protein from the smallest and simplest known viruses capable of autonomously replicating in animal cells, circoviruses, to establish structural and mechanistic insights into capsid morphogenesis and regulation. The beak and feather disease virus, like many circoviruses, encode only two genes: a capsid protein and a replication initiation protein. The capsid protein forms distinct macromolecular assemblies during replication and here we elucidate these structures at high resolution, showing that these complexes reverse the exposure of the N-terminal arginine rich domain responsible for DNA binding and nuclear localization. We show that assembly of these complexes is regulated by single-stranded DNA (ssDNA), and provide a structural basis of capsid assembly around single-stranded DNA, highlighting novel binding interfaces distinct from the highly positively charged N-terminal ARM domain.

## Abstract (web summary)

Circoviruses are the simplest viruses known to autonomously replicate in vertebrates. Here the authors present three structures for distinct macromolecular assemblies of the capsid protein from the beak and feather disease virus that provides insights into the regulation of viral capsid assembly.

## Graphical abstract

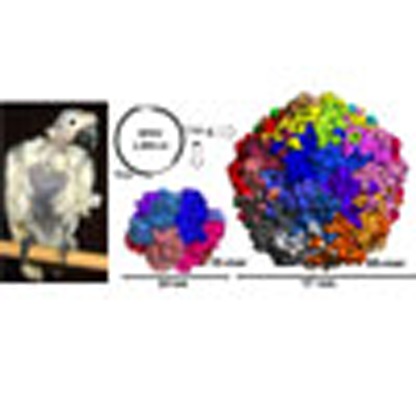

The assembly of viral capsid proteins into large macromolecular complexes is essential for viral replication. Circoviruses, the smallest and simplest of all viruses known to autonomously replicate in vertebrates, represent models of biological efficiency, harbouring only two genes encoded within a two kilobase (kb) single-stranded DNA (ssDNA) genome. During replication, the capsid protein forms distinct assemblies ranging from large non-enveloped spherical capsid virions with icosahedral symmetry to smaller complexes localized in the cytoplasm and nucleus^1^,^2^,^3^,^4^. How disparate and complex molecular assemblies associate from single viral proteins remains to be resolved at a molecular level, particularly for assemblies that switch between icosahedral and non-icosahedral symmetry and present different functional modalities. Here, we present three high-resolution X-ray crystallographic structures for distinct macromolecular assemblies of the capsid (*Cap*) protein from the beak and feather disease virus (BFDV), a circovirus infecting critically endangered parrots. These complexes include a 10 nm assembly, resolved at 2.0 Å and comprised of two face-to-face pentamers of the *Cap* molecules, and two 17 nm assemblies comprised of 60 *Cap* monomers in the absence and presence of ssDNA, determined to 2.5 and 2.3 Å resolution respectively in the same space group, arranged as 12 pentamers. These assemblies exhibit distinct monomeric and pentameric units, and unique inverted morphologies that reverse the accessibility of the DNA binding and nuclear localization signal (NLS) domains present within the N-terminal arginine rich motif (ARM), important for viral assembly. We also show that assembly is highly influenced by ssDNA, and have elucidated cryo electron microscopy (cryoEM) and crystal structures of the capsid protein bound to ssDNA, identifying an unexpected DNA-binding interface. Our results provide unique insights into a regulation mechanism of viral capsid morphogenesis based on ssDNA recognition that couples assembly and genome packaging.

## Results

### Structural characterization of capsid assemblies

To carry out genome replication, most DNA viruses invade the nucleus of a host cell to utilize polymerases and other host enzymes. Circoviruses, harbouring as few as two genes, lack an autonomous DNA polymerase and depend on the high-fidelity host machinery for *de novo* DNA synthesis by rolling circle replication^5^. However, unlike many non-enveloped animal DNA viruses such as parvoviruses, adenoviruses and polyomavirus which replicate in the nucleus with accumulated mature virus particles released by karyolysis or apoptotic pathways, pathogenic circoviruses cause large globular intracytoplasmic inclusions composed of paracrystalline virus arrays (Supplementary Fig. 1). In addition to these paracrystalline array assemblies, circovirus capsid proteins have been shown to exist in multiple conformational assemblies during replication, including small intracytoplasmic non-membrane bound assemblies of 0.1–0.5 μm, larger membrane bound inclusion bodies of 0.5–5.0 μm, intranuclear inclusion bodies composed of circular virus complexes of 10–12 nm, as well as fully mature, single icosahedral virus like particles (VLPs) of 17 nm (refs 1, 2, 3, 4). These assemblies reflect in part the multiple functions performed by *Cap*, some of which extend beyond its structural role. Once internalized, *Cap* recruits host dynein/microtubule machinery to travel through the cytoplasm towards the nuclear membrane^6^. Passage of viral molecules across the nuclear pore complex occurs via protein importation routes directed by NLSs in the flexible ARM domain of *Cap* which are recognized by host receptors of the importin family and other cofactors^7^. *Cap* also interacts with the replicase associated protein^8^, mediates DNA binding, and can self-associate to form a range of intracellular assemblies^1^,^2^,^3^,^4^,^7^.

**Figure 1:**
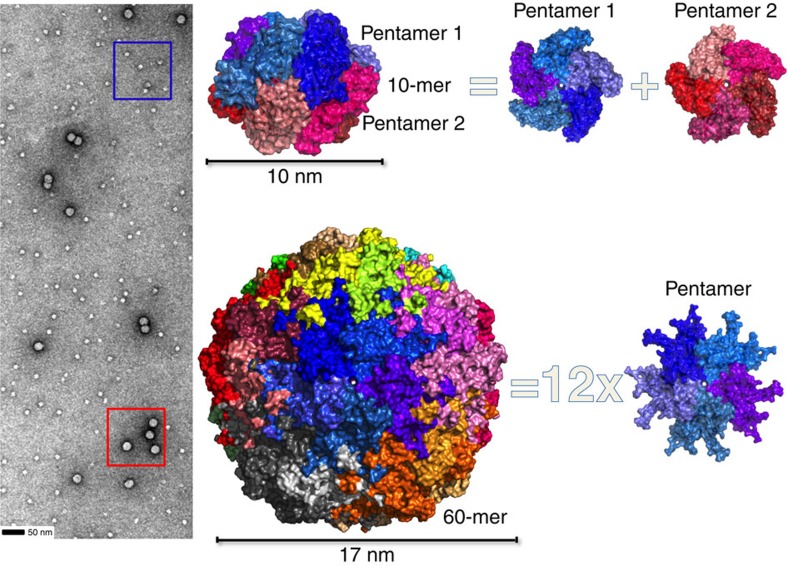
Structural characterization of two distinct BFDV-*Cap* complexes. Left panel, negatively stained electron micrograph of the BFDV *Cap* protein shows two populations corresponding to VLPs (red box), and a smaller assembly of ∼10 nm in diameter (blue box). Right panel, X-ray crystal structures allow modelling of the two complexes to 2.0 Å (10 nm, top), and 2.5 Å (17 nm, bottom). The smaller complex is comprised of 10 *Cap* molecules arranged as two interlocking discs, with each disc containing five *Cap* molecules. The larger VLP is comprised of 12 pentamers arranged with *T*=1 icosahedral symmetry.

**Figure 2:**
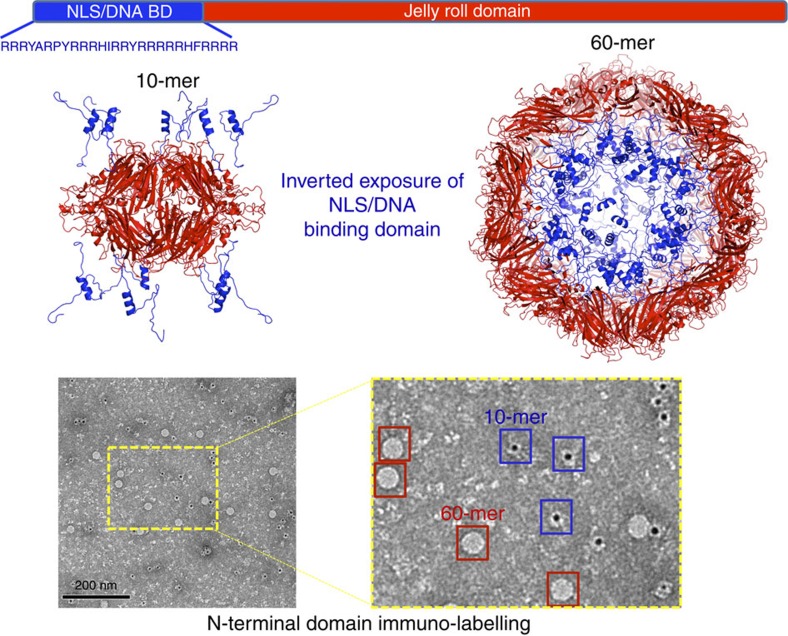
The N-terminal ARM domain accessibility is inverted in *Cap* complexes. X-ray crystal structures reveal that the N-termini of all *Cap* molecules are positioned on the exterior and interior of the 10- and 60-mer complexes respectively (top panel). The accessibility of the highly positively charged N-terminal ARM domains containing both NLS and DNA-binding activity, modelled using I-tasser^12^ in blue cartoon, is confirmed in electron microscopy immunogold-labelling experiments of the N-terminal domain.

To better understand the capacity of capsid proteins to form different assemblies, the BFDV-*Cap* protein was recombinantly expressed, purified and confirmed by EM and atomic force microscopy (AFM) to form both VLPs of ∼17 nm and the smaller assembly of ∼10 nm, matching those observed during infection^1^,^2^,^3^,^4^ (Fig. 1 and Supplementary Fig. 2). To resolve these complexes at atomic resolution, a combination of crystallography and EM approaches were employed. X-ray diffraction to 2.0 Å enabled the atomic coordinates of the 10 nm complex to be elucidated. Ten capsid molecules with D5-symmetry were present in the asymmetric unit (ASU; Fig. 1, Table 1) (ref. 9), and all capsid monomers within the ASU were structurally equivalent, with the greatest root mean square deviation (r.m.s.d.) between any two monomers <0.5 Å. Each *Cap* monomer was comprised of a canonical viral jelly roll^10^ built of two, four-stranded antiparallel β-sheets, with sheet one comprised of β-strands B, I, D, G, and the other sheet comprised of β-strands C, H, E, F (Supplementary Fig. 3). These monomers associate tightly to form two pentamers in a face-to-face orientation, analogous to two interlocking discs (Fig. 1). The assembly buries an extensive surface area of 13,650 Å^2^, of which 60% is associated between monomers contained within each pentamer, and 40% buried at the interface between the two pentamers (Supplementary Fig. 4). Monomers within each pentamer are arranged radially around a central pore of ∼7 Å, with Tyr^115^ exposed at the surface of the pore, and Val^117^ lining the inner surface (Supplementary Fig. 5). Each monomer within a pentamer associates with two adjacent monomers, burying 1,660 Å^2^ of surface area, and these interactions are mediated through salt bridge (SB) interactions and an extensive hydrogen bonding (HB) network (Supplementary Table 1, Supplementary Fig. 4). Interactions between the two pentamers are also extensive, with every *Cap* monomer forming interactions with two *Cap* molecules in adjacent pentamers, burying 655 and 415 Å^2^ of surface area at each interface. These interactions are predominantly mediated through hydrogen bond (HB) interactions (Supplementary Table 1). The overall diameter of the decameric assembly is 10 nm, and encloses an internal volume^11^ of 306,078 Å^3^, ∼1/7 the volume of the fully mature virus capsid assembly (see below). Interestingly, the N-termini of every *Cap* monomer is positioned towards the exterior of the assembly, and therefore the highly positively charged N-terminal ARMdomains (^14^IRRRYARPYRRR HIRRYRRRRRHF^37^), crucial for DNA binding and association with nuclear import receptors, are highly accessible as modelled using I-tasser^12^ in Fig. 2. This contrasts strongly with the 60-mer *Cap* assembly where all N-termini are buried within the interior of the virus (see below and Fig. 2). The ability of the virus to present these functional domains is important for localizing *Cap* to the nucleus, allowing co-localization with newly replicated viral ssDNA, as well as interaction and packaging of the viral DNA. Premature formation of icosahedral VLPs (see ‘Discussion' section) would internalize and mask these domains, causing mis-localization and segregation of the capsid from its ssDNA genome. The ability of ssDNA to influence the equilibrium between these two species is also presented below.

The crystal structure of the second assembly identified in electron micrographs (Fig. 1) and infected tissue^1^,^2^,^3^,^4^ was resolved to 2.3 Å and comprised of sixty capsid molecules arranged as twelve pentamers (Fig. 1). Five capsid proteins were present in the ASU, with the unit cell comprised of 480 *Cap* proteins arranged as eight icosahedral VLPs. The assembly buries a total surface area of 182,920 Å^2^ (compared with 13,650 Å^2^ in the 10-mer), with a notable shift in the ratio of intra- and inter-pentamer interactions, the majority now occurring between pentamers (68% involved in inter-pentamer interactions compared with 40% in the decamer). The interactions that occur within the pentamer are comparable to the 10 nm assembly, with each capsid burying 1,934 Å^2^, mediated through 22 HB and 4 SB (compared with 1,660 Å^2^ for the 10-mer assembly). Each pentamer interacts with five other pentamers, and every *Cap* molecule within a pentamer interacts with three other *Cap* molecules from other pentamers. Two sets of these interactions are identical and together bury 3,390 Å^2^ (through 52 HBs, 4 SBs; Extended Data Table 1), and the third interaction site buries 774 Å^2^ (mediated through eight HBs, Supplementary Table 1). The diameter of the VLP is 17 nm, corresponding to the size of the infectious particle, and exhibits a volume of 2,171,590 Å^3^. Structural comparison with the porcine circovirus 2 (PCV2) VLP, the only other circovirus VLP structure to be determined to date, reveals an r.m.s.d of 2.19 Å over 210 residues^13^ between the monomeric units, a slightly reduced internal volume of 2,034,684 Å^3^ of the VLP, less buried surface area of 141,240 Å^2^ (compared with 182,920 Å^2^ of the BFDV VLP) and a reduction in the number of bonds that mediate VLP formation (1,020 for PCV2; 2,700 for BFDV; see Extended Data Table 1 for full interactions). The most striking differences lie at the centre of the 3-fold axis symmetry, where in BFDV, two insertions of eight and four residues (EDLTTANQ_182_; GGPN_203_) create protruding loops from the VLP and extensive interactions (see Supplementary Table 1 for complete list of interactions). Thus while these axes have been reported to create a valley in the PCV2 VLP (ref. 13), these regions represent some of the most protruded areas in the BFDV VLP.

**Figure 3:**
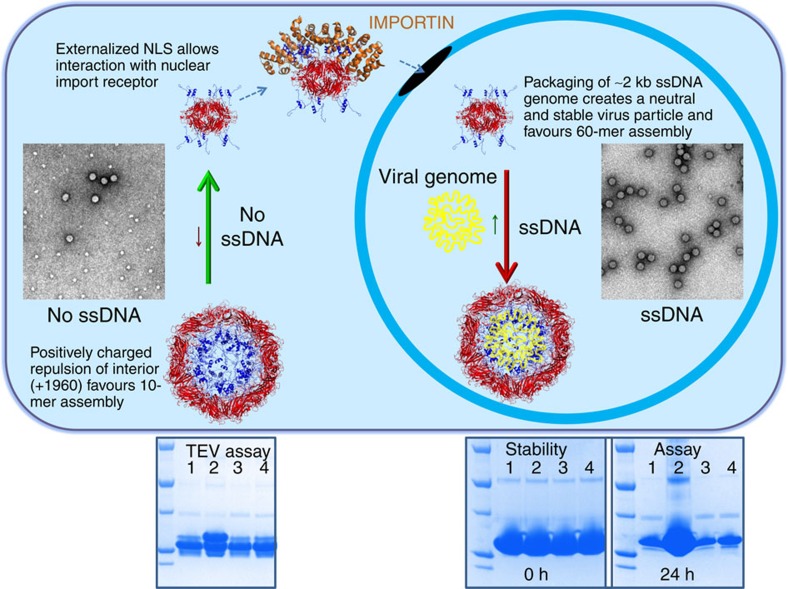
The population of the two viral complexes is regulated by ssDNA. Negatively stained images of *Cap* complexes (see electron micrograph inserts in top panel) reveal 10-mer complexes are highly favoured in the absence of ssDNA, while ssDNA promotes 60-mer particle assembly. This is highly intuitive for the localization of *Cap* to the nucleus, as well as its interaction and packaging of the ssDNA viral genome. Synthesis of *Cap* molecules, occurring in the cytoplasm which is segregated from its ssDNA genome, require an accessible NLS and DNA-binding domains for interaction with nuclear import receptors and viral ssDNA respectively. Under these conditions, the 10-mer complexes are favoured over the 60-mer, which if formed in the cytoplasm would render these functional domains inaccessible, inhibiting nuclear localization and interaction with ssDNA. This is supported by Tobacco Etch Virus (TEV) and stability Assays (bottom panels), showing that in the absence of ssDNA (lane 1), the N-termini are exposed and susceptible to TEV proteolysis (left panel), and that the protein is less stable (right panel). Premature assembly of empty VLPs are limited in the absence of ssDNA due to the strong repulsive forces of the positively charged ARM domains. In the presence of ssDNA (lane 2), but not double-stranded DNA (lane 3) or plasmid DNA (lane 4), 60-mer particles are strongly favoured (see micrograph inserted in top panel). In the nucleus, the presence of ssDNA promotes the formation of 60-mer particles that are more stable, with the negatively charged ssDNA neutralizing the charge of the positively charged N-terminal ARM domains.

Since both BFDV *Cap* assemblies are comprised of pentameric protomers, we tested whether these units could be computationally interchanged within respective assemblies. Superimposition of pentamers within the biological unit of each complex revealed that neither pentamer could substitute within the respective assembly, moreover superimposition of individual *Cap* proteins also produced major steric clashes within respective complexes (Supplementary Fig. 6). Key differences within the *Cap* molecules reside predominantly within the loop regions of the smaller β-sheet jelly roll domain (CHEF), spanning residues 42–46, 77–97, 126–153 and 166–211 (Supplementary Fig. 6). Differences in buried surface area between the two assemblies, 182,920 Å^2^ for the 60-mer, and 13,650 Å^2^ for the 10-mer, suggests that the 60-mer should be highly favoured; however, analysis by AFM and EM shows a higher proportion of the 10-mer complexes in the absence of ssDNA (Fig. 3). We proposed that this is due to a destabilizing effect caused from the repulsion of the highly positively charged ARM domains present within the 60-mer when ssDNA is not packaged (see Figs 2 and 3; overall charge of the virus interior is +1,960 in the absence of ssDNA). To test whether the presence of ssDNA could stabilize the assembly of a full virus capsid, we compared the assemblies in the absence and presence of a ssDNA oligonucleotide. We found that in the presence of ssDNA, 60-mer particles were highly favoured with 10-mer complexes almost non-existent in electron micrographs (Fig. 3). This transition may be highly informative for virus assembly; the synthesis of the *Cap* and newly synthesized viral ssDNA occurs in cytoplasm and nucleus respectively, thus segregated by the nuclear envelope. Premature assembly of the 60-mer *Cap* particles in the cytoplasm would produce empty VLP's and also mask the NLS and DNA-binding domains, preventing co-localization and packaging of viral ssDNA in the nucleus. Thus, in the absence of DNA, *Cap* preferentially forms 10-mer complexes, where the highly accessible ARM domains can mediate nuclear localization and DNA binding (Fig. 3). Once *Cap* is localized to the nucleus, the presence of the ssDNA viral genome favours the 60-mer assembly since the strong positively charged repulsive forces are neutralized by binding the ssDNA viral genome. Interestingly, the overall positive charge of the N-terminal *Cap* molecules in the 60-mer assembly (+1,960) is almost exactly equal to the ssDNA charge of the viral genome (∼2 kb ssDNA genome), creating a neutral and comparatively stable particle (Fig. 3). This is supported in our stability assays (Fig. 3, bottom panels), and consistent with other studies demonstrating that nucleotide binding promotes spontaneous formation of VLPs (ref. 13).

### Structural basis of BFDV-Cap interaction with ssDNA

**Figure 4:**
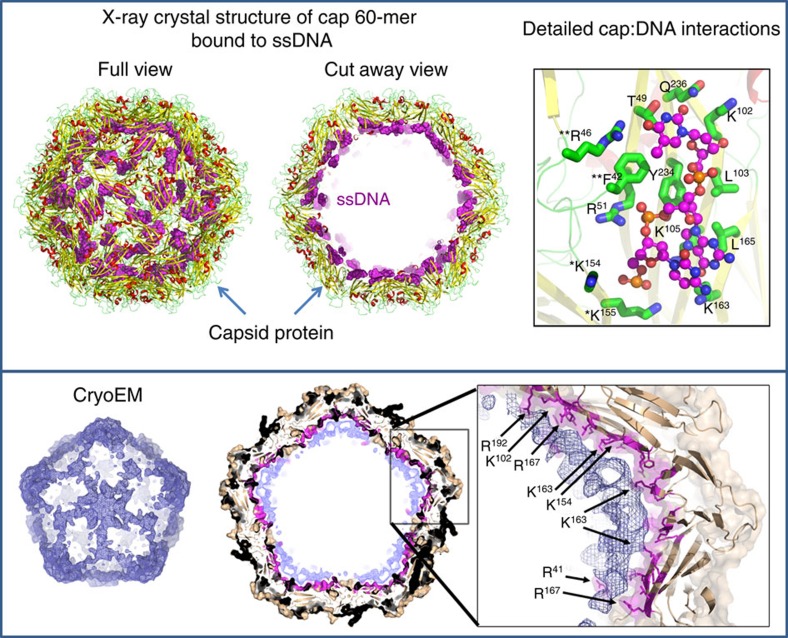
X-ray crystal and cryoEM structures of the *Cap*:ssDNA complex reveal novel ssDNA binding sites. Top panel: Crystal diffraction of the *Cap*:ssDNA complex to 2.3 Å enabled modelling of 180 nucleotides (spheres) on the interior of the capsid (cartoon). Detailed interactions are presented in the top right panel, involving a range of side-chain interactions (presented as sticks). Residues listed with * and ** are from adjacent *Cap* monomers within and external to a pentameric protomer, respectively. A cryoEM reconstruction of the Cap:ssDNA complex (bottom panel) to 4.5 Å revealed density supporting the modelled ssDNA in the crystal structure, contiguous to the centre of the VLP (bottom panel). Electron density corresponding to the capsid protein is coloured in brown. The surface of the capsid interacting with the DNA is coloured in magenta. The remaining electron density is represented as a blue mesh both in the difference map (bottom left panel) and the full reconstruction (middle panel).

Analysis of the electrostatic charges on the interior and exterior surfaces of the particles revealed a highly positively charged interior surface, and a possible additional interface for DNA binding (Supplementary Fig. 7). These positively charged surfaces are distinct from the positively charged ARM domains, and mediated through dense clusters of internalized Lys and Arg residues (R_46_, R_51_, R_100_, K_102_, K_105_, R_109_, K_154_, K_155_, R_160_, K_163_, R_164_, R_167_, K_169_, K_230_). To determine the precise mechanism of DNA binding, we co-crystallized the BFDV-*Cap* protein in the presence of ssDNA labelled with AlexaFluor488 and AlexaFluor647. Crystals diffracting to 2.3 Å formed in the presence of labelled DNA were highly fluorescent (Supplementary Fig. 8), and displayed clear positive difference density (Supplementary Fig. 8) corresponding to ssDNA. The capsid*-*DNA complex, modelled and refined to an R-factor/Free R-factor (R-work/Rfree) of 0.173 and 0.197, respectively, revealed an extensive array of electrostatic interactions with each ssDNA chain interacting with residues T_49_, R_51_, K_102_, L_103_, K_105_, K_163_, L_165_, Y_234_, Q_236_ of one *Cap* chain, K_154_, K_155_ of an adjacent chain within the pentamer, and F_42_, and R_46_ of an adjacent, inter-pentamer chain (Fig. 4). That each ssDNA chain spans three *Cap* monomers, both within and across pentameric protomers is consistent with the increased stability observed in our assays (Fig. 3). These DNA-binding residues are highly conserved in BFDV genomes (Supplementary Fig. 9). In total, 180 nucleotides are modelled on the interior surface of the capsid, and cryoEM data suggests that the remaining unmodelled ssDNA would occupy the interior of the virus capsid assembly, thus shielding the positively charged ARM domains (Fig. 4).

Our results provide detailed, high-resolution structural insightsinto ssDNA-mediated regulation of viral *Cap* assembly into distinct complexes with inverted functional domains. This has important implications for rationalizing antibodies against N-terminal domain fragments previously reported in the literature, but not structurally resolved^13^. Understanding these complexes also provides a platform for the delivery of novel therapies, with the decameric structure representing the smallest viral nano-cage (Supplementary Fig. 10), exhibiting highly desirable properties including a charged interior surface capable of binding small interfering RNA sequences, and exposure of the N-terminus providing a plethora of cell specific tags to be engineered to direct viral assemblies.

## Methods

### Cloning, expression and purification

The target gene encoding BFDV-Cap residues 14–247 was amplified from a plasmid containing the BFDV entire genome (KF385406) and cloned into the pMCSG21 expression vector by ligation independent cloning using the specific primers; forward primer **TACTTCCAATCCAATGCC**AGACGACGATATGCCCGCCCA and reverse primer **TTATCCACTTCCAATGTTA**TTAAGTACTGGGATTGTTAGGGGCAAAC where the bolded nucleotides are required for the ligation independent cloning procedure^14^ and the underlined nucleotides are complementary to the gene sequence. This construct encodes an N-terminal 6-his tag, TEV protease site, and residues encoding the BFDV-Cap protein. The fidelity of the construct was confirmed by DNA sequencing and the recombinant plasmid overexpressed in *Escherichia coli* BL21 (DE3) Rosetta 2 cells (Novagen, USA). A 5 ml Luria–Bertani (LB) starter culture containing 100 μg ml^−1^ spectinomycin was used to inoculate 500 ml of LB expression media. The cells were grown at 37 °C to an OD_600_ of 0.6, the temperature lowered to 25 °C, and protein expression induced by addition of 1 mM isopropyl-β-D-1-thiogalactopyranoside (IPTG; Sigma). Following expression for 12 h at 25 °C, the cells were collected by centrifugation at 6,000 r.p.m. for 30 min and the cell pellet resuspended in buffer A, containing 20 mM N-cyclohexyl-3-aminopropanesulfonic acid, pH 10.5, 500 mM NaCl, 30 mM imidazole, pH 10.5 and stored at −20 °C. The bacterial cells were lysed by two repetitive freeze-thaw cycles in the presence of 20 mg lysozyme, 0.5 mg of DNaseI and FastBreak Cell Lysis buffer (1X; Promega). Lysates were centrifuged at 15,000 r.p.m. for 30 min at 4 °C, and the supernatant filtered through a 0.45 μm low protein-binding filter and applied to a 5 ml Ni^2+^ column (HisTrap HP, GE Healthcare) preequilibrated with buffer A. Following extensive washing of the column (>10 column volumes) the protein was eluted using an increasing gradient of buffer B (20 mM N-cyclohexyl-3-aminopropanesulfonic acid, pH 10.5, 500 mM NaCl and 500 mM imidazole, pH 10.5). Elution fractions were pooled and further purified by size exclusion chromatography (Superdex 200 column, GE healthcare) in GST-A containing 20 mM Tris pH 8.0, 125 mM NaCl. The peak fractions were pooled and concentrated to 15 mg ml^−1^ using an Amicon ultrafiltration device (Millipore), the purity assessed by SDS–PAGE to be >95% pure, aliquoted and stored at −80 °C.

### Crystallization

Crystallization experiments were performed using the hanging drop vapour diffusion method and screened using commercially available screens (PEG/Ion, PEG/Ion 2, Crystal Screen, Crystal Screen 2, ProPlex and PACT premier) in VDX 48-well plates from Hampton Research. The crystallization drops consisted of 1.5 μl of protein solution mixed with 1.5 μl of reservoir solution, suspended above 300 μl of reservoir solution and incubated at 290 K. Small crystals obtained from the screens were optimized by varying the precipitant, buffer, salt concentrations and additives. The final crystallization conditions for the 10-mer contained 1.1 M ammonium sulfate, Tris (pH 8.5) and 0.1 M iron chloride; final crystallization conditions for the 60-mer contained 0.8 M Na/K hydrogen phosphate (pH 7.4). Crystals formed in the presence of HPLC purified ssDNA contained the fluorescent labelled dye at the 3' end and following sequences (BFDVRepAlexaFlour488: 5′-CCGGACGCAAAATGAAGGAAGTCGCGCGAGAGTTCCC-3′; BFDVRepAlexaFlour647: 5′-CGCGGTGACCGTCTCTCGCCACAATGCCCA-3′) purchased from Sigma-Aldrich, USA.

### Data collection and structure determination

All crystals were collected and cryoprotected in the well reservoir solution containing 25% glycerol and immediately flash-cooled in liquid nitrogen. All diffracted data were collected at the Australian Synchrotron on the MX2 macromolecular crystallography beamline. All data sets were indexed, integrated and scaled using the program iMOSFLM (ref. 15) and scaled with Aimless from the ccp4 suite^16^,^17^. The structure was solved by molecular replacement with the program Phaser^9^ using a modified model of residues 46 to 246 of the PCV2 capsid virion from PCV2 (ref. 13) as a search model (∼30% sequence identity). Refinement and model building was performed in Phenix refine^18^ and COOT, respectively (see Table 1)^19^. Lack of electron density for the N-terminal 6-His tag and ARM domains precluded modelling of these regions and were not present in the final structure.

### Stability and TEV cleavage assay

Each reaction contained purified BFDV-*Cap* (360 μg) mixed with 2 mM of ssDNA, dsDNA and ds-plasmid DNA in a final volume of 30 μl. In the absence of DNA, the volume of the reaction mixture was adjusted with respective buffer. The stability of the BFDV-*Cap* proteins were assessed by centrifugation to remove precipitated proteins, and the supernatant analysed by SDS–PAGE after 0 and 24 h incubation at room temperature. Tobacco etch virus (TEV) assays were performed by treating the samples with 5 μl of TEV (3.3 mg ml^−1^), and assessed by SDS–PAGE after 1 h incubation.

### Negative stain and immuno-labelling electron microscopy

For negative staining, samples were applied to glow-discharged carbon-coated grids and stained with 2% aqueous uranyl acetate. For immunogold labelling, complexes were applied to glow-discharged carbon-coated grids and the grids were blocked with TNE buffer (50 mM Tris–HCl pH 7.5, 150 mM NaCl, 5 mM EDTA), 5% normal goat serum, 1% BSA. Monoclonal αHis serum was incubated for 45 min, followed by three washes with TNE, 0.5% normal goat serum, 0.1% BSA. The samples were then incubated with αMouse antibody conjugated to 5-nm gold particles for 1 h, washed and negatively stained as above. Images were recorded on a 1 k Gatan CCD camera in a Tecnai 12 FEI microscope operated at 120 kV. Samples containing ssDNA contained the following synthetically produced oligonucleotide: 5′-CGCGGTGACCGTCTCTCGCCACAATGCCCA-3′.

### Transmission electron microscopy

Feather samples for transmission electron microscopy were fixed in 5% glutaraldehyde in phosphate buffer with 1% calcium chloride (200:1) and stored for 2 h at room temperature. Fixed tissues were washed in Sorenson's buffer and covered with Dalton's Chrome Osmic Acid for 1.5 h at 4 °C. The samples were then dehydrated with 70, 90, 95 and 100% ethanol then in two changes of propylene oxide over 15 min. Samples were placed in propylene oxide/EPON 812 (60:40) for 1 h at 4 °C before being embedded in capsules for 24 h at 6 °C. Ultra-thin sections were cut at 90 nm and placed on 200 mesh copper grids, stained with uranyl acetate for 5–7 min, washed and then stained with lead citrate for 4 min. Sections were washed thoroughly by dipping the grid in distilled water then allowed to dry on clean filter paper.

### Atomic force microscopy

Measurements were performed with an AFM (Nanotec Electrónica, Madrid, Spain) operating in Jumping mode plus (Ortega-Esteban), using force-versus-Z-piezo-displacement curves at every point after a nanometric lateral displacement of the tip when it is far from the sample. Rectangular silicon-nitride cantilevers (RC800PSA, Olympus, Center Valley, PA) with a nominal spring constant of 0.05 N m^−1^ were used and calibrated by Sader's method^20^, thus allowing to take images at low forces (between 60 and 90 pN). The experiments were carried out in a liquid medium composed of 5 μl of BFDV complexes with or without ssDNA (at a concentration of 1 mg ml^−1^ in 50 mM Tris, 125 mM NaCl), diluted in 45 μl of 50 mM Tris, 125 mM NaCl to a final concentration of 0.1 mg ml^−1^. Each sample was incubated for 15 min on a fresh highly ordered pyrolytic graphite surface (ZYA quality; NT-MDT, Tempe, AZ) and washed with buffer until a volume of 100 μl was reached. The tip was also prewetted with a 30 μl drop of buffer before image acquisition. Images were processed using the WSxM software^21^.

### Cryo electron microscopy

Five microlitres of sample was applied to a glow-discharged holey carbon grid (Quantifoil R1.2/1.3) for preparing frozen-hydrated specimen using a Vitrobot Mark IV (FEI) with a 3 s blotting time at 100% humidity. Grids were transferred under liquid nitrogen to a Titan Krios transmission EM (FEI) operated at 300 kV and set for parallel illumination. One second exposures with a calibrated magnification of ∼127, 000 (corresponding to a pixel size of 1.02 Å on the specimen) were automatically recorded on a Falcon 2 camera (FEI) in movie mode using a dose rate of 45 electrons per second controlled by data acquisition software EPU (FEI). The corresponding 17 sub-frames were fractionated in 7 frames as follows: Sub-frame 1 was discarded. Sub-frames 2–7 were recorded as frames 1–6, respectively. Sub-frames 8–16 were pooled and integrated as frame 7. Sub-frame 17 was discarded. The defocus was set to a range of 0.6 μm to 3.5 μm in intervals of 0.2 μm.

### Image processing

Movies were integrated in EMAN 2 (ref. 22) by averaging all seven frames. RELION 1.4 (ref. 23) was used as wrapper for CTF estimation with CTFFIND3 (ref. 24) and for evaluating integrated images for astigmatism and drift. Approximately 60,000 particles were automatically selected from a small subset of the remaining images using the swarm tool of the e2boxer.py program of EMAN2. Two dimensional classification of this initial data set in RELION 1.4 provided class averages that served as templates for particle selection across all retained images using the autopick function in RELION 1.4. Particles were extracted with a box size of 400 × 400 pixels and subjected to another round of 2D classification in RELION 1.4 yielding high-quality class averages. Three representative classes were subjected to the e2initialmodel.py program in EMAN2 for generating an initial model. The initial model was low-pass filtered to 60 Å and provided to 3D classification in RELION 1.4 for disentangling the full data set into homogenous subsets. Particles assigned to the best 3D class were further refined using the ‘gold-standard' approach in RELION 1.4. Beam-induced movements were corrected by movie processing and particle polishing only extracting frames 1–6 of the movies and without a shifting particles average. Resolution of the final reconstructions was determined using the gold-standard Fourier Shell Correlation (FSC) criterion: FSC=0.143. The pixel size was determined to be 1.02 Å by optimization of the fit of the crystal structure of Cap into the cryoEM reconstruction using Chimera. A difference map between the fitted crystal structure of Cap and the cryoEM reconstruction was computed in Chimera.

### Data availability

Structures described in this manuscript have been deposited in Protein Data Bank under accession code 5J09, 5J36, and 5J37 for the 10-mer, 60-mer and 60-mer+ssDNA structures, respectively. The cryo-EM data has been deposited and issued the code EMDB-8306. The authors declare that all other data supporting the findings of this study are included in the manuscript and its Supplementary Files or are available from the corresponding author on request.

## Additional information

**How to cite this article:** Sarker, S. *et al*. Structural insights into the assembly and regulation of distinct viral capsid complexes. *Nat. Commun.* **7,** 13014 doi: 10.1038/ncomms13014 (2016).

## Supplementary Material

Supplementary Information Supplementary Figures 1-10 and Supplementary Table 1

## Figures and Tables

**Table 1:** Data collection and refinement statistics.

	***Cap*** **10-mer 5J09**	***Cap*** **60-mer 5J36**	***Cap*** **60-mer:ssDNA 5J37**
*Data collection*			
Space group	*P* 2_1_ 2_1_ 2_1_	F 4 3 2	F 4 3 2
Cell dimensions			
*a*, *b*, *c* (Å)	78.8, 148.4, 188.6	377.3, 377.3, 377.3	377.3, 377.3, 377.3
*α, β, γ* (°)	90, 90, 90	90, 90, 90	90, 90, 90
Resolution (Å)	30–2.0 (2.03–2.0)*	34–2.55 (2.60–2.55)	40–2.3 (2.34–2.30)
*R*_pim_	0.043 (0.241)	0.075 (0.438)	0.067 (0.416)
*I/*σ(*I*)	10.7 (2.9)	7.3 (1.6)	7.5 (1.6)
*CC*_1/2_	0.99 (0.87)	0.99 (0.68)	0.99 (0.70)
Completeness (%)	99.2 (98.0)	99.9 (99.8)	100.0 (100.0)
Redundancy	4.4 (4.1)	9.1 (7.1)	8.6 (5.9)
			
*Refinement*			
Resolution (Å)	30–2.0 (2.07–2.00)	34–2.55 (2.64–2.55)	40–2.3 (2.38–2.30)
No. unique reflections	149,556 (14,483)	74,754 (7,344)	101,169 (9,985)
*R*_work_	0.2079 (0.2462)	0.1912 (0.2542)	0.1729 (0.2430)
*R*_free_	0.2385 (0.2879)	0.2162 (0.2861)	0.1970 (0.2743)
No. atoms	15,272	8,662	9,275
Protein	13,865	8,310	8,135
Ligand ssDNA	NA	NA	480
Ligand PO_4_	NA	25	25
Water	1,407	327	635
*B* factors	28.09	35.35	32.5
Protein	27.54	35.25	32.4
Ligand ssDNA	NA	NA	66.0
Ligand PO_4_	NA	97.9	45.8
Water	33.6	33.0	34.0
*R.m.s. deviations*			
Bond lengths (Å)	0.002	0.002	0.003
Bond angles (°)	0.58	0.57	0.64
*Ramachandran plot (%)*			
Favoured	98	98	98
Allowed	2	2	2
Outliers	0	0	0
Rotamer outliers (%)	1.1	0.78	0.56
Clashscore	1.12	0.60	0.88

NA, not applicable; r.m.s, root mean square; ssDNA, single-strand DNA.

^*^Values in parentheses are for highest-resolution shell.
