# Supplementary material for: Structural insights into the assembly and regulation of distinct viral capsid complexes
Source: Nat Commun. 2016 Oct 4;7:13014. doi: 10.1038/ncomms13014 (PMC5059447; doi:10.1038/ncomms13014)
Supplement: Supplementary Information — Supplementary Figures 1-10 and Supplementary Table 1 [file ncomms13014-s1.pdf]

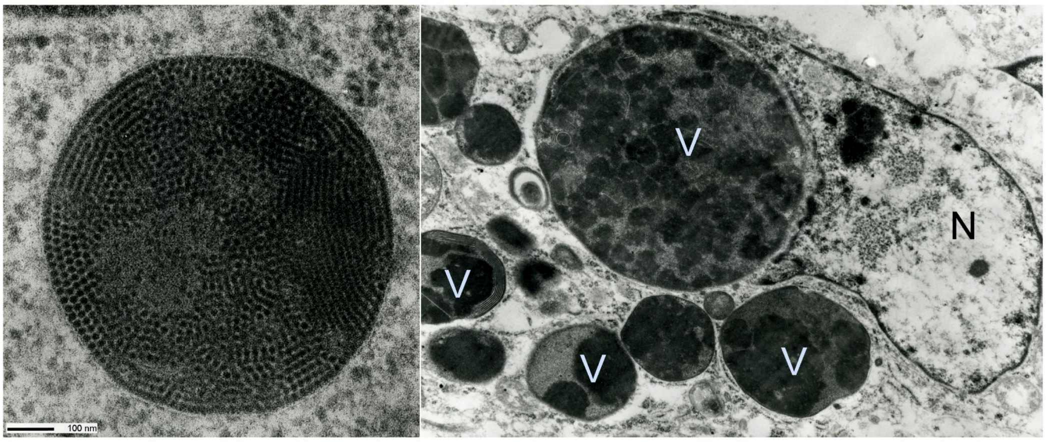

Supplementary Fig. 1. Transmission electron micrograph of BFDV infected cell. Right panel demonstrating how the nucleus (N) is relatively spared, with large crystalline arrays of mature virus particles preferentially forming intra-cytoplasmic inclusions (V) shown at higher magnification on the left.

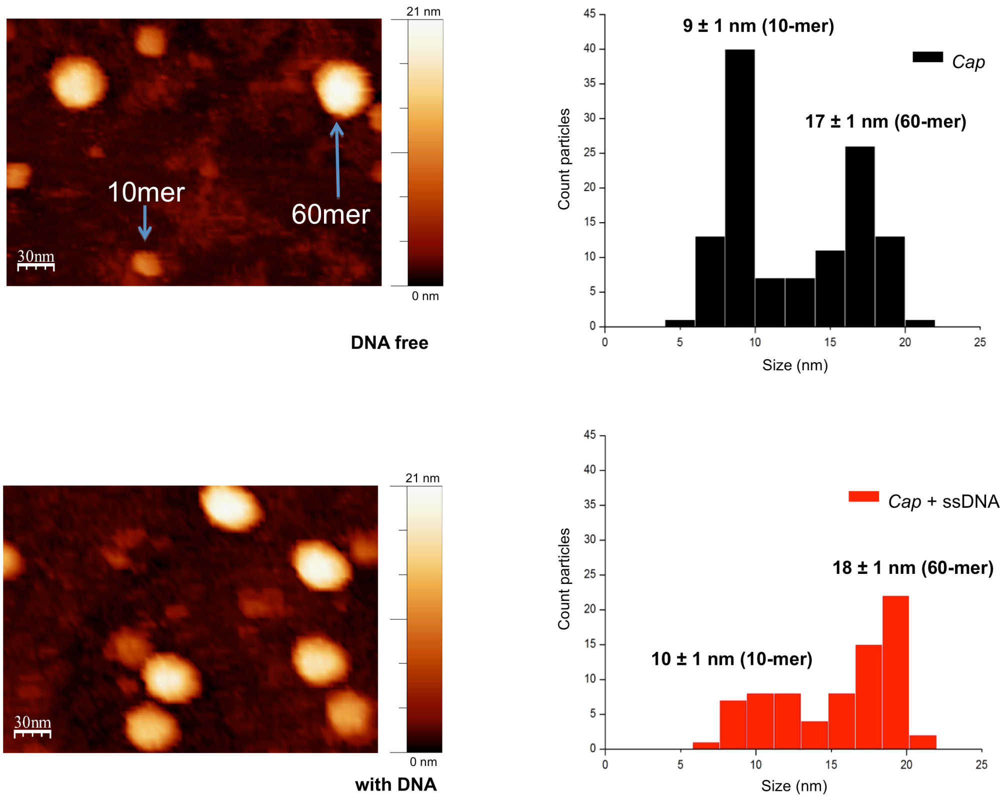

Supplementary Fig. 2. AFM of *Cap* complexes show two populations. Left panels, representative AFM images of BFDV *Cap* complexes without (top left) and with (bottom left) ssDNA. Right panels, histograms of the complex sizes. The two clearly defined population sizes for samples without ssDNA (top right) are 17 ± 1 (mean ± standard deviation) nm and 9 ± 1 nm (n = 119), and for samples with ssDNA (bottom right) there is one predominant population with a size of 18 ± 0.4 (n = 75).

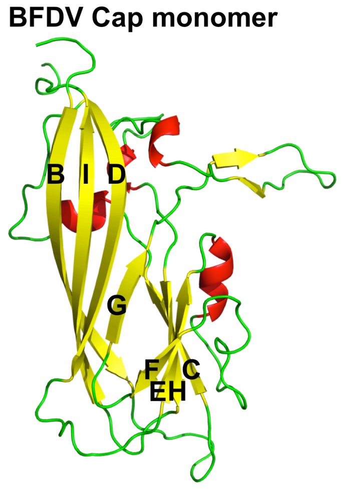

Supplementary Fig. 3. The *Cap* protein monomer forms a jelly roll domain. The monomer is comprised of two β-sheets, the larger of which is comprised of β-strands B, I, D, and G, and the smaller comprised of β-strands C, H, E, and F. Strands are depicted in cartoon and colored yellow, loops are colored green, and helices in red.

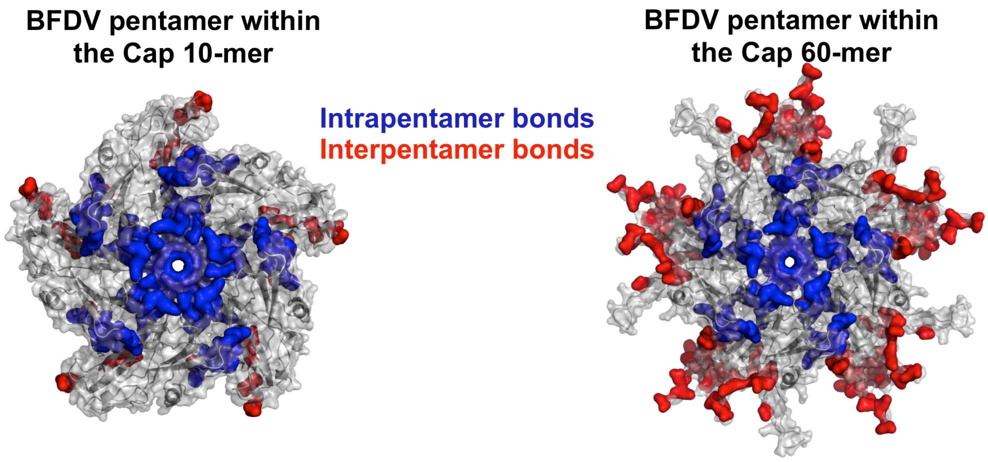

10-mer

FSTNRIYTLRLTRQFQFKINKQTTSVGNLIFNADYITFALDDFLQAVPNPHTLNFEDYRIKLA
KMEMRPTGGHYTVQSDGFGHTAVIQDSRITRFKTTADQTQDPLAPFDGAKKWFVSRGFKRLLR
PKPQITIEDLTTANQSAALWLNSARTGWIPLQGGPNSAGTKVRHYGIAFSFPQPEQTITYVTK
LTLYVQFRQFAPNNPST

60-mer

FSTNRIYTLRLTRQFQFKINKQTTSVGNLIFNADYITFALDDFLQAVPNPHTLNFEDYRIKLA
KMEMRPTGGHYTVQSDGFGHTAVIQDSRITRFKTTADQTQDPLAPFDGAKKWFVSRGFKRLLR
PKPQITIEDLTTANQSAALWLNSARTGWIPLQGGPNSAGTKVRHYGIAFSFPQPEQTITYVTK
LTLYVQFRQFAPNNPST

Supplementary Fig. 4. Association of *Cap* monomers into pentamers and higher order complexes are mediated through an extensive array of contacts. The pentameric protomers of the 10-mer (left) and 60-mer (right) are colored by residues that mediate intra- and inter-pentamer interactions in blue and red respectively. The bottom sequence panels depict the specific residues involved in these interactions.

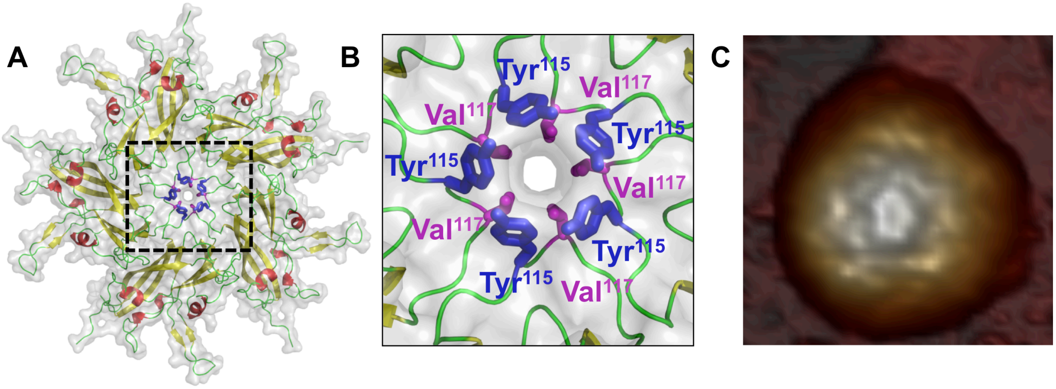

Supplementary Fig. 5. The five-fold symmetry axis forms a pore. (A) Crystal structure depicted as a cartoon model and (B) zoomed view of the pore formed through interactions of five *Cap* proteins. The pore, lined by Tyr^{115} and Val^{117} residues, is approximately 7 Å in diameter. (C) AFM image of the 5-fold axis.

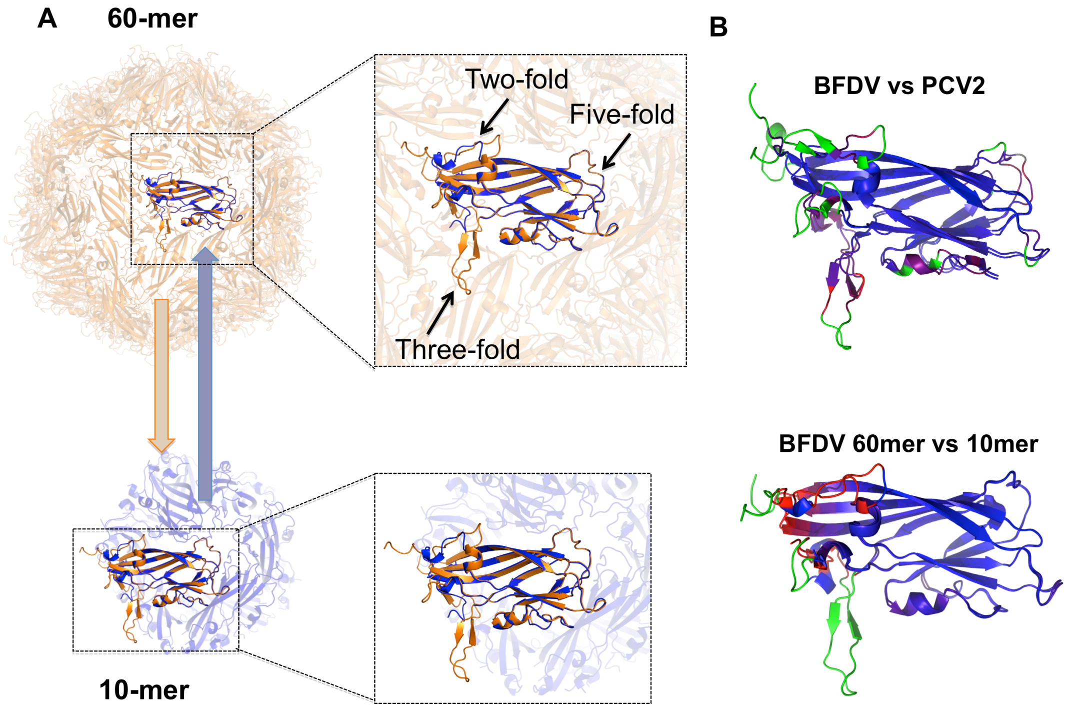

Supplementary Fig. 6. The *Cap* proteins cannot substitute within the respective complexes. A) Superposition of a *Cap* molecule from the 60-mer (orange) onto a *Cap* monomer within the 10-mer (blue) reveals a range of steric clashes at residues 42, 43, 44, 46, 48, 50, 102, 186, 187, 190, 192, 239, 240; whilst superposition of a *Cap* monomer (blue) in a 60-mer particle results in steric clashes at residues 41, 49, 52, 86, 89, 105, 143, 146, 149, 151, 164, 192. These clashes are predominantly localized to the two loop regions that mediate interactions at the two- and three-fold axis of the *Cap* 60-mer particles. B) Superposition of monomeric units within the BFDV and PCV2 VLPs (top panel), and monomer units within the BFDV 60-mer and 10-mer complexes. Coloring highlights the r.m.s.d between structures, with blue representing low r.m.s.d and high structural similarity, red representing high r.m.s.d and poor structural similarity, and green regions highlighting regions that could not be superimposed due to loop insertions or unmodelled regions.

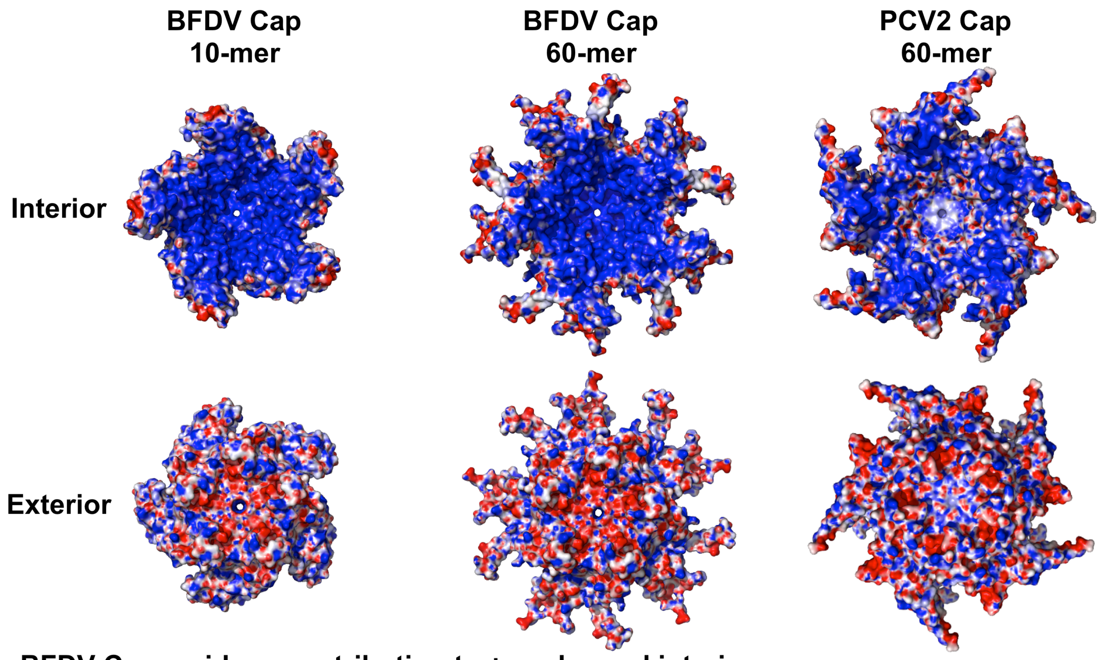

BFDV Cap residues contributing to +ve charged interior

FSTN**R**IYTL**R**LTRQFQFKINKQTTSVGNLIFNADYITFALDDFLQAVPNPHT
LNFEDY**R**I**K**LA**K**MEM**R**PTGGHYTVQSDGFGHTAVIQDSRITRFKTTADQTQD
PLAPFDGA**KK**WFVS**R**GF**KR**LL**R**P**K**PQITIEDLTTANQSAALWLNSARTGWIP
LQGGPNSAGTKVRHYGIAFSFPQPEQTITYVT**K**LTLYVQFRQFAPNNPST

Supplementary Fig. 7. The inner surface of the *Cap* complexes is highly positively charged. Surface representation colored by electrostatic residues are depicted on the interior and exterior of the two *Cap* particles. Residues responsible for the highly positively charged interior are highlighted in blue in the sequence below.

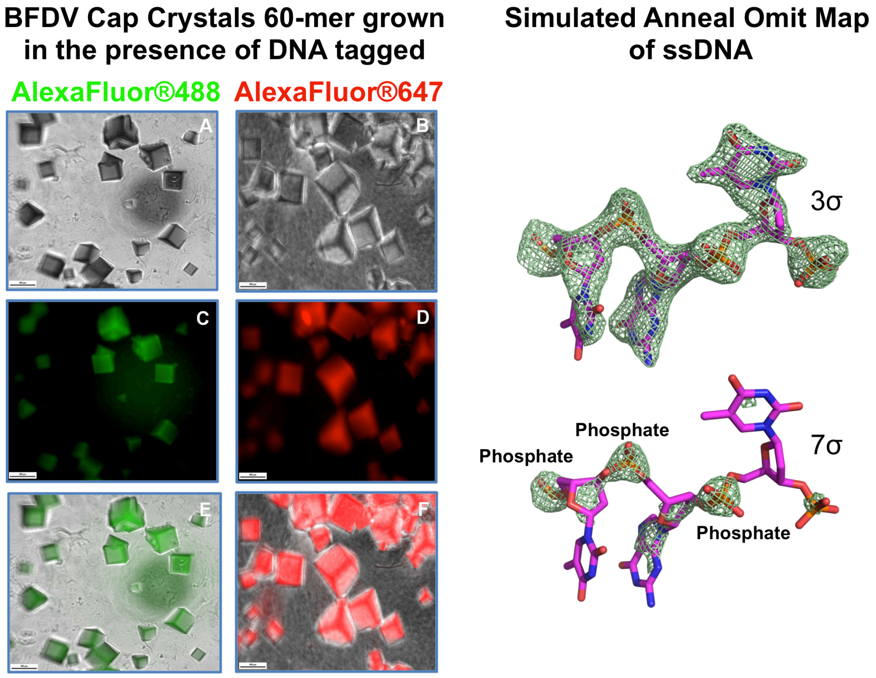

Supplementary Fig. 8. Crystal of *Cap* grown in the presence of labelled ssDNA are highly fluorescent. BFDV *Cap* crystals grown in the presence of DNA labelled with Alexa Fluor® 488 (a bright green-fluorescent dye; left panel) and Alexa Fluor® 647 (a bright far-red fluorescent dye; right panel). A & B) BFDV-*Cap* crystals with DNA labelled with Alexa Fluor® 488 and Alexa Fluor® 647, viewed under white light. C & D, crystals viewed under UV light, and E & F merged image of the above fields. Right panel. The ssDNA superimposed onto the positive density of a simulated annealed omit map at 3 σ (top) and 7 σ (bottom) respectively. The latter contour level highlights the strong phosphate diffraction within the ssDNA nucleic acid.

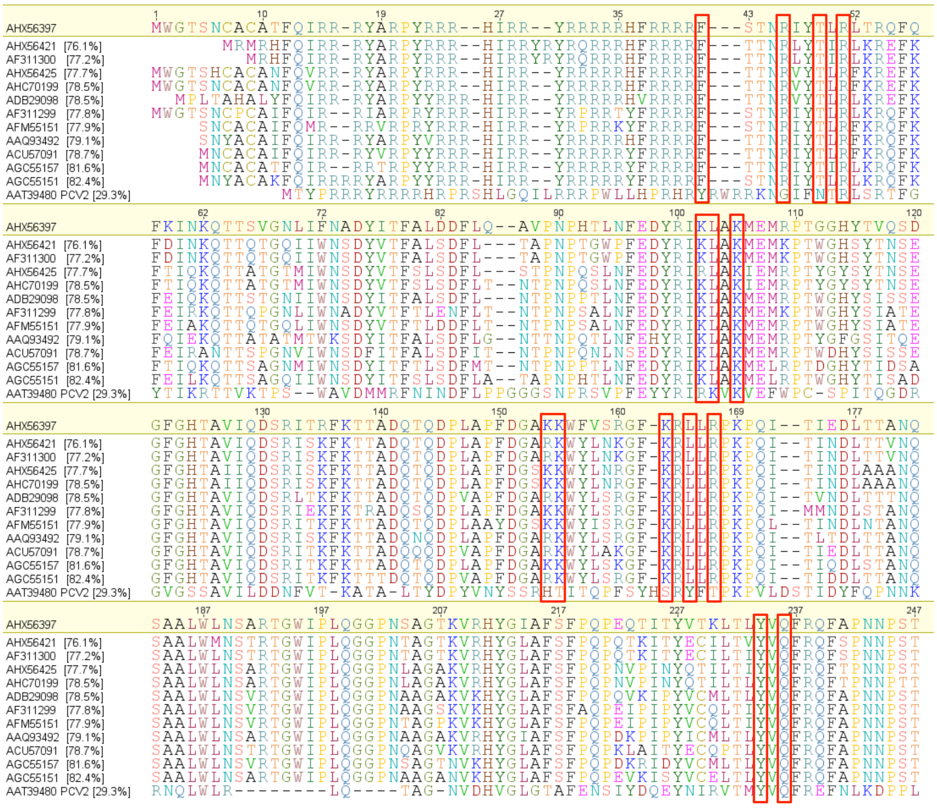

Supplementary Fig. 9. Residues mediating ssDNA binding on the inner capsid surface are highly conserved. BFDV *Cap* protein sequences exhibiting the highest divergence were selected from 446 full *Cap* sequences available in GenBank. The sequence similarity between two representative sequences of a BFDV *Cap* (AHX56397) and a PCV2 *Cap* (3r0r) is 30%. Red box highlights conserved residues of BFDV *Cap* that interact with ssDNA in the crystal structure.

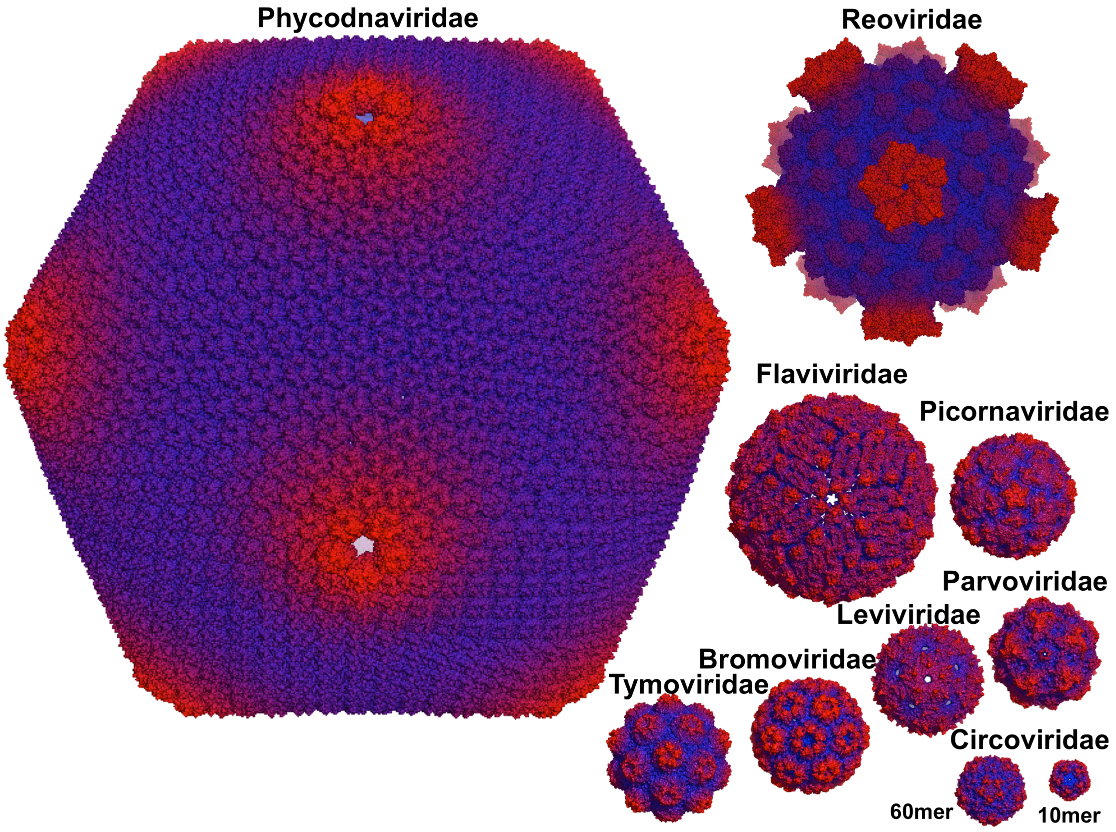

Supplementary Fig. 10. **The circoviruses are the smallest of all known viruses known to autonomously replicate in animal cells**. Surface views of the Chlorovirus (Phycodnaviridae, PDB 1M4X, 1,694 Å diameter); Reovirus (Reoviridae, PDB 1EJ6, 734 Å diameter); Dengue virus (Flaviviridae, PDB 1K4R, 504 Å diameter); Rhinovirus (Picornaviridae, PDB 1AYM, 310 Å diameter); Turnip Yellow Mosaic Virus (Tymoviridae, PDB 1AUY, 300 Å diameter); Brome Mosaic Virus (Bromoviridae, PDB 1JS9, 282 Å diameter); Bacteriophage MS2 (Leviviridae, PDB 2MS2, 276 Å diameter); Canine parovirus (Parvoviridae, PDB 2CAS, 276 Å diameter); and the Beak and Feather Disease Circovirus Capsid particles of the 60-mer VLP, 170 Å in diameter, and the 10-mer complex, 100 Å in diameter.

Supplementary Table 1. Intra- and inter-molecular interactions within the Cap 10-mer and 60-mer assemblies of BFDV and PCV2 VLP
***Italics represent salt bridge interactions**

BFDV Cap 60-mer

Intra-pentamer interfaces x 60
Each surface buries 967 Å^2; 11 HB; *2 SB*

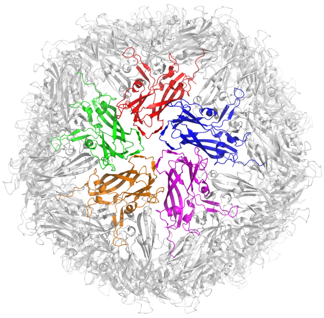

GLY 68[O]	2.38	GLN 57[HE22]
VAL117[O]	1.95	THR116[H]
VAL117[O]	3.36	THR116[OG1]
SER119[O]	2.33	GLY113[H]
SER119[H]	2.05	HIS114[O]
GLY121[H]	2.48	PRO110[O]
GLN144[OE1]	2.10	GLN 55[H]
GLN144[HE22]	1.87	GLN 55[O]
ASP145[H]	2.01	GLN 55[OE1]
PHE150[O]	2.31	ARG 51[HH21]
LYS155[HZ2]	1.83	GLU107[OE2]
LYS155[NZ]	*2.99*	*GLU107[OE1]*
LYS155[NZ]	*2.71*	*GLU107[OE2]*

Inter-pentamer interfaces x 60
Each surface buries 1,695 Å^2; 26 HB; *2 SB*

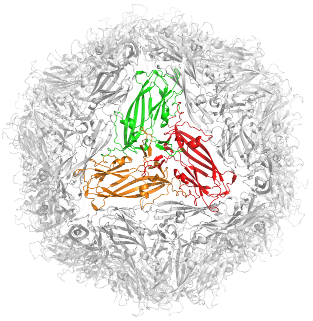

VAL127[O]	1.99	GLN239[HE22]
GLN129[OE1]	2.34	ARG238[HH22]
GLN129[OE1]	2.47	ARG238[HH12]
ARG132[HE]	1.95	ASN244[OD1]
ARG132[HH11]	2.10	GLU 97[OE1]
ARG132[HH21]	2.18	ASN244[OD1]
ARG132[HH22]	2.01	PRO242[O]
GLN142[HE22]	2.01	SER246[O]
GLN142[OE1]	2.28	SER246[H]
GLN142[OE1]	3.14	SER246[OG]
ASP151[H]	2.00	PHE240[O]
ASP151[OD2]	1.99	PHE240[H]
ASP151[OD2]	1.79	TYR 48[HH]
GLY152[H]	2.20	PHE 42[O]

PCV2 Cap 60-mer

Intra-pentamer interfaces x 60
Each surface buries 720 Å^2; 6 HB; 0 SB

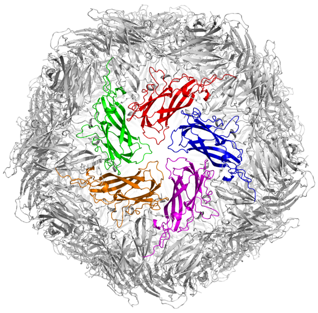

ARG111[H]	2.16	CYS103[O]
ARG111[HE]	2.29	SER104[O]
ARG111[HH12]	1.95	PHE149[O]
ARG111[O]	2.38	CYS103[HG]
ASN138[OD1]	2.17	SER 45[H]
GLU198[OE1]	2.19	ASN207[HD21]

Inter-pentamer interfaces x 60
Each surface buries 1077 Å^2; 10 HB; 1 SB

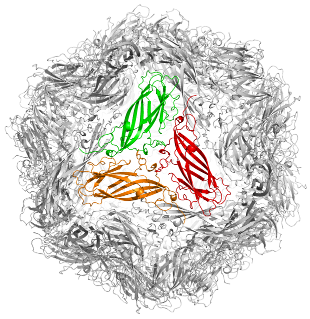

ASN123[HD22]	2.05	GLU 89[OE1]
LEU120[H]	2.02	TYR 90[OH]
PHE169[H]	2.25	TRP179[O]
SER141[H]	2.12	GLU218[OE2]
ASN138[HD22]	2.20	LEU221[O]
TYR139[OH]	2.27	LYS222[HZ1]
SER140[OG]	2.22	PHE219[H]
THR165[O]	2.23	GLN183[HE22]
ASP167[OD2]	2.36	ARG181[HE]
PHE169[O]	2.02	TRP179[H]
ASP167[OD2]	*3.13*	*ARG181[NE]*

THR173[O]	2.30	THR173[H]
GLU175[OE2]	3.52	THR173[OG1]
ASP176[O]	2.17	ARG210[H]
ASP176[OD1]	1.87	TYR212[HH]
ASP176[OD2]	2.11	ARG210[HH11]
THR178[H]	2.09	LYS208[O]
THR178[OG1]	3.61	LYS208[O]
SER183[H]	2.26	ASN203[OD1]
TRP187[H]	2.28	TRP195[O]
TRP187[O]	2.05	TRP195[H]
ASN189[H]	2.06	THR193[O]
ASN189[HD22]	2.22	ARG192[O]
ARG132[NH1]	*2.87*	*GLU 97[OE1]*
ASP176[OD2]	*2.78*	*ARG210[NH1]*

Inter-pentamer interface x 30
Each buries 774 Å^2; 8 HB; *0 SB*

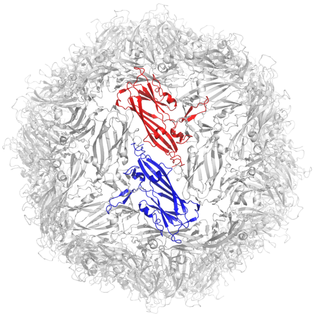

ARG 46[HH21]	2.13	THR 49[O]
ASN243[HD22]	2.14	VAL 88[O]
ASN243[H]	1.87	PRO 89[O]
ASN243[HD21]	2.04	ASN 90[O]
THR 49[O]	1.97	ARG 46[HH21]
VAL 88[O]	2.23	ASN243[HD22]
PRO 89[O]	1.91	ASN243[H]
ASN 90[O]	2.03	ASN243[HD21]

Inter-pentamer interface x 30
Each buries 1114 Å^2; 0 HB; *0 SB*

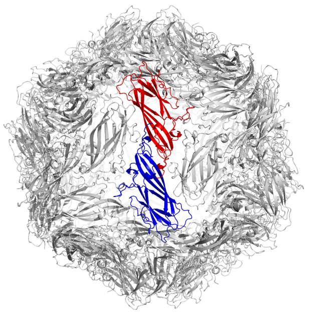

Cap 10-mer

Intra-pentamer interfaces x 10
Each buries 830 Å^2; 10 HB; *2 SB*

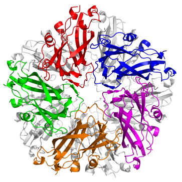

GLN 57[HE22]	2.04	GLY 68[O]
THR116[H]	2.06	VAL117[O]
GLY113[H]	2.03	SER119[O]
GLN 55[H]	2.12	GLN144[OE1]
ARG 51[HH22]	2.36	ALA148[O]
GLN 55[OE1]	1.83	ASP145[H]
GLN 55[O]	1.98	GLN144[HE22]
GLU107[OE1]	2.24	LYS155[HZ2]
PRO110[O]	2.49	GLY121[H]
HIS114[O]	1.79	SER119[H]
GLU107[OE1]	*3.01*	*LYS155[NZ]*
GLU107[OE2]	*3.01*	*LYS155[NZ]*

Inter-pentamer interfaces x 5
Each buries 655 Å^2; 5 HB; *0 SB*

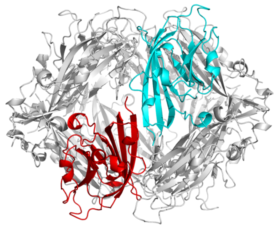

THR 49[H]	2.10	THR 49[O]
GLN239[HE22]	1.79	ASN 90[O]
THR 93[OG1]	3.60	GLN239[OE1]
THR 49[O]	2.14	THR 49[H]
THR 93[O]	1.81	ASN 95[HD22]

Inter-pentamer interface x 5
Each buries 415 Å^2; 3 HB; *4 SB*

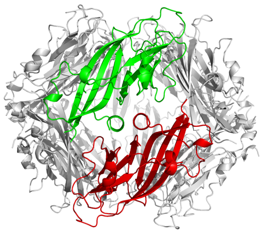

ASP151[OD1]	2.30	LYS102[HZ1]
ASP151[OD1]	1.99	LYS102[HZ2]
ASP151[OD2]	2.31	GLN236[HE22]
LYS102[NZ]	*3.28*	*ASP151[OD2]*
ARG100[NH2]	*3.91*	*ASP151[OD2]*
ASP151[OD1]	*2.53*	*LYS102[NZ]*
ASP151[OD2]	*3.27*	*LYS102[NZ]*
